# Neonatal *Chlamydia muridarum* respiratory infection causes neuroinflammation within the brainstem during the early postnatal period

**DOI:** 10.1186/s12974-024-03150-3

**Published:** 2024-06-15

**Authors:** Kateleen E Hedley, Henry M Gomez, Eda Kecelioglu, Olivia R Carroll, Phillip Jobling, Jay C Horvat, Melissa A Tadros

**Affiliations:** 1 School of Biomedical Sciences & Pharmacy, The University of Newcastle Callaghan, NSW, 2308 Australia; 2 Hunter Medical Research Institute, New Lambton Heights, NSW Australia

**Keywords:** Medulla oblongata, Cytokines, Microglia, Astrocytes, Sex-specific

## Abstract

Respiratory infections are one of the most common causes of illness and morbidity in neonates worldwide. In the acute phase infections are known to cause wide-spread peripheral inflammation. However, the inflammatory consequences to the critical neural control centres for respiration have not been explored. Utilising a well characterised model of neonatal respiratory infection, we investigated acute responses within the medulla oblongata which contains key respiratory regions. Neonatal mice were intranasally inoculated within 24 h of birth, with either *Chlamydia muridarum* or sham-infected, and tissue collected on postnatal day 15, the peak of peripheral inflammation. A key finding of this study is that, while the periphery appeared to show no sex-specific effects of a neonatal respiratory infection, sex had a significant impact on the inflammatory response of the medulla oblongata. There was a distinct sex-specific response in the medulla coincident with peak of peripheral inflammation, with females demonstrating an upregulation of anti-inflammatory cytokines and males showing very few changes. Microglia also demonstrated sex-specificity with the morphology of females and males differing based upon the nuclei. Astrocytes showed limited changes during the acute response to neonatal infection. These data highlight the strong sex-specific impact of a respiratory infection can have on the medulla in the acute inflammatory phase.

## Introduction

There is a critical window of development that ranges from birth to adolescence where the periphery and central nervous system (CNS) undergo major changes. During this time, the periphery and CNS are especially sensitive to insults, and in particular acute inflammatory events [1]. Acute respiratory tract infections are one of the most common causes of peripheral inflammation, resulting in significant morbidity and mortality in neonates worldwide, with up to 18% of Australian children admitted to hospital with respiratory distress within the first two years of life [2]. One of the major causes of respiratory tract infections in neonates is *Chlamydia trachomatis* [3]. *C. trachomatis* is passed onto an infant through vertical transmission during childbirth, resulting in a severe respiratory infection with lung deficits, wheeze and increased neutrophilic infiltration [4]. Older infants are more likely to contract *Chlamydia pneumonia* via community transmission, which results in a similar respiratory phenotype of lung dysfunction and significant inflammation. Neonatal peripheral inflammatory events have been linked to respiratory dysfunction later in life, such as asthma [5], however not all aspects of these conditions can be explained by peripheral respiratory deficits alone. The CNS has been proposed as a contributor to respiratory dysfunction [6] and this dysfunction is driven by the nuclei that control respiration, housed in the medulla oblongata of the brainstem [7]. However, the specific neuroinflammatory impacts of a neonatal respiratory infection on these brainstem centres have not been explored.

There are many respiratory regions within the medulla oblongata forming a circuit that drives respiration, however there are three primary centres that are critical to basic function of the respiratory system. Inflammation from the lungs is thought to affect these nuclei via the vagus nerve, via a number of well described pathways that ultimately end with the release of inflammatory mediators [8] within the nuclei that receive and supply information from the vagus nerve. These are the nucleus tractus solitarii (NTS), a major integration and dissemination centre for sensory information entering the CNS through the vagus nerve and the dorsal motor nucleus of the vagus (DMX), providing parasympathetic pre-ganglionic innervation to the smooth muscle in the bronchi. The inflammatory mediators stimulated at these nuclei then go on to interact with microglia and astrocytes within the CNS, causing a cascade of neuroinflammation [1].

The inflammatory cascade has been shown to be detrimental, not only in the acute phase but also leading to long-term changes. The neonatal period has been shown to be particularly suspectable to neuroinflammation, with a pro-inflammatory response that appears to be regulated by microglia [9] and increased susceptibility to changes in blood brain barrier (BBB) permeability that could allow infiltration of peripheral immune cells into the CNS [10]. This has linked neonatal neuroinflammation to several neurological disorders [11], as well as causing disruption to the local CNS environment, which can alter neuronal signalling and result in peripheral organ dysfunction, for example the lungs and heart [12].

In this study, utilising our well-established model of neonatal respiratory infection using *Chlamydia muridarum* [13, 14], we aim to provide evidence that a neonatal respiratory infection does acutely affect the respiratory centres in the medulla oblongata, by altering the inflammatory profile in a sex-specific manner. We showed altered inflammatory mediators within the CNS, including cytokines and chemokines, as well as modulation of microglial activity, the resident immune cell of the CNS, with limited changes in astrocytes, that maintain the BBB. Intriguingly, these changes occur in a sex-specific manner, even during this neonatal period.

## Methods

### Animals

All experiments were performed in accordance with the National Health and Medical Research Council Australian Code of Practice and as approved by the University of Newcastle Animal Care and Ethics Committee (approval number A-2020-028). Pregnant BALB/c mice were acquired from the University of Newcastle’s Central Animal House, with the pups born to these mothers used as the experimental animals. Animals were housed under specific pathogen free (SPF), PC2 conditions in individually ventilated cages (Bioresources facility, HMRI Building, New Lambton Heights, NSW, Australia). All animals were maintained in a temperature (22 °C ± 2) and humidity-controlled environment on a 12 h light/dark cycle with food (standard chow) and water available ad libitum for the duration of the study.

### Neonatal *Chlamydia muridarum* respiratory infection model

The laboratory model of neonatal *Chlamydia muridarum* (CMU) has been previously published [14]. Briefly, within 24 h of birth, (assigned postnatal day 0 [P0]), neonatal BALB/c mice were infected intranasally with either *Chlamydia muridarum* (CMU, 400 inclusion-forming units, ATCC VR-123, in 5µL sucrose phosphate glutamate buffer [SPG]) or sham-infected with SPG alone. Mice were weighed and rate of weight gain was calculated (g/d) throughout the experiments as a measure of health status. Mice were then sacrificed at P15 (peak of peripheral inflammation [13]) and tissues collected as outlined below. A total of 55 offspring were used for this study from 13 litters (SPG derived from 6 litters, CMU derived from 7 litters). Each individual offspring was considered an individual experimental unit. Mothers were used for one litter only. Molecular and anatomical studies used both males and females from SPG and CMU groups, with each litter being evenly distributed across both techniques. Both males and females were assessed independently, thus experimental groups are as follows; female SPG (FSPG), female CMU (FCMU), male SPG (MSPG) and male CMU (MCMU).

### RNA extraction and RT-qPCR of lung tissue

For confirmation of infection, lungs at P15 were assessed for the presence of 16 S, confirming bacteria within the lungs, and a selection of inflammatory markers. Following euthanasia with sodium pentobarbital (150 mg/kg intraperitoneally [i.p.]), the lungs (multi-lobe, *n* = 7–8 per group) were removed and immediately snap-frozen to -80 ^O^C in liquid nitrogen. The RNA was extracted using standard protocols [15]. Briefly, the tissue was thawed and homogenised (1 mL of TRIzol, 4 °C; ThermoFisher Scientific, Australia) with a tissue tearor homogeniser (Biospec Products, Bartlesville, OK, USA). Chloroform was added and then centrifuged (12,000× g, 15 min, 4 °C) to precipitate out the DNA, and the aqueous phase containing the RNA was collected. With the addition of isopropyl alcohol, the RNA was precipitated out and pelleted (12,000× g, 10 min, 4 °C), then washed twice with 75% ethanol. The RNA pellets were dissolved in nuclease-free water and RNA concentration and quality were measured with a NanoDrop 1000 Spectrophotometer (ThermoFisher Scientific, USA).

For cDNA synthesis, any contaminating genomic DNA remaining in the RNA samples was digested using DNase I (Sigma-Aldrich, Macquarie Park, Australia; 15 min, room temperature) and then the samples were heated (10 min, 65 °C). Random hexamer primers (50 ng, Meridian Bioscience, Memphis, TN, USA) and dNTPs (10 mM, Meridian Bioscience, Memphis, TN, USA) were added and samples were further heated (5 min, 65 °C). Dithiothreitol (10 mM) and Bioscript reverse transcriptase (200 units in reaction buffer, Meridian Bioscience, Memphis, TN, USA) were added, and reverse transcription was performed (10 min, 25 °C; 50 min, 42 °C; 15 min, 70 °C).

All qPCR primers (Table 1) were designed with Ensembl using the standard primer design criteria. The primer pairs were then put through NCBI primer BLAST to ensure primer specificity. A total volume of 12 µl was used containing: 6 µl iTaq Universal SYBR Green Supermix (Bio-rad, NSW, Australia), 10µM each of forward and reverse primers, 2000ng cDNA and molecular grade water to 3 µl. After an initial 2 min 60 °C enzyme activation step, 40 cycles of 95 °C for 15 s (step 1) followed by 60 °C for 30 s (step 2) were completed. Melt curves were generated to confirm the presence of a single PCR product. Primers were deemed specific if a single amplified product of appropriate size was detected by melt curve analysis. Reactions were performed on a Bio-Rad CFX384 Touch Real-Time PCR System (Bio-Rad, NSW, Australia) and analysed using the Bio-Rad CFX Manager (version 3.1). For each primer the samples were run on a 384-well plate in triplicate, including a negative water control on each plate. Delta Ct (ΔCt, threshold cycle) was determined for each gene relative to the housekeeping gene, hypoxanthine-guanine phosphoribosyl transferase (HPRT), and the well-established ΔΔCt method [16] was employed to enable comparisons between the groups.

**Table 1:** Forward and reverse primer sequences

Gene	Forward Primer (5’->3’)	Reverse Primer (5’->3’)
Β-Actin	GCAGGAGTACGATGAGTCCG	ACGCAGCTCAGTAACAGTCC
18s	ACCAGACTTGCCCTCCAATG	AACGGCTACCACATCCAAGG
HPRT	AGGCCAGACTTTGTTGGATTTGAA	CAACTTGCGCTCATCTTAGGCTTT
16 S	GCGGCAGAAATGTCGTTTT	CGCTCGTTGCGGGACTTA
IL-1β	GAAGTTGACGGACCCCAAAA	GCCTGCCTGAAGCTCTTGTT
TNF⍺	GCCTCTTCTCATTCCTGCTTG	CTGATGAGAGGGAGGCCATT
NLRP3	GTGGATGGGTTTGCTGGGAT	CCTGCTTCTCACATGTCGTCT
CXCL9	CAGAGCCAGACAGGGTGAAA	AGCAGTCCCAAATATCCGCA
CXCL10	AAGCTATGTGGAGGTGCGAC	TGAGCTAGGGAGGACAAGGA
IL-13	AGACTCCCCTGTGCAACGGCA	GGAGACCGTAGTGGGGGCCTT
KCNN4	CCAGTCAGGAGGCCACATAG	AGAAAACACAGGAGCAGGGAT
Stat1	CAGAAAAACGCTGGGAACAGA	GGTGGTCTCCAGGTCAATCA
Stat6	TCCACGAGCTTCACATTGGG	AGGATGGTAGCTTTGGCGTT
TGFβ	CTGCTGACCCCCACTGATAC	AGCCCTGTATTCCGTCTCCT
PTGS2	AATACTGGAAGCCGAGCACC	AAGAAGTGAAGGGACACCCC
BDNF	CCTGCATCTGTTGGGGAGAC	TGGTCATCACTCTTCTCACCTG
IFN𝛾	CTGGAGGAACTGGCAAAAGG	TTGCTGATGGCCTGATTGTC

### RNA extraction and RT-qPCR of medulla oblongata

Brainstems (*n* = 7–8 per group) were removed following euthanasia (i.p. sodium pentobarbital 150 mg/kg) and immediately snap-frozen to -80 ^O^C in liquid nitrogen. The medulla oblongata was then separated from the whole brainstem, using the cerebral peduncles and the dorsal median sulcus as landmarks, and homogenised. Total cellular RNA was extracted using the RNeasy® Mini Kit (Qiagen, Hilden, Germany), following manufactures instructions. RNA concentration and quality were measured with a NanoDrop 1000 Spectrophotometer (ThermoFisher Scientific, USA).

Any contaminating genomic DNA remaining in the RNA samples was digested using DNase I (Invitrogen, Scoresby, Australia). Reverse transcription was then performed using Superscript III (Invitrogen, Scoresby, Australia), according to manufacturer’s instructions. Briefly, 30–200ng of total RNA, 1 µl of oligo(dT)_18_ (Meridian Bioscience, Ohio, USA), 1 µl of random hexar (Meridian Bioscience, Ohio, USA), 1 µl of 10µM dNTP (Meridian Bioscience, Ohio, USA), and molecular grade water to 13 µl, were mixed and heated for 5 min at 65 °C in a Thermal Cycler (Eppendorf, Germany). Next, 4 µl of 5x first-strand buffer, 1 µl of 0.1 M DTT, 1 µl RNaseOUT (40 U/µl) (Meridian Bioscience, Ohio, USA) and 1 µl SuperScript III RT (200 U/µl) were added and the mixture was incubated for 60 min at 50 °C, followed by 70 °C for 15 min. Reverse transcription without Superscript III was also performed to assess genomic DNA contamination.

The same qPCR primers (Table 1) were used and the protocol was similar to that of the lungs, with the exceptions detailed below. A total volume of 12.5 µl was used containing: 6.25 µl 2x SensiFAST SYBR (Meridian Bioscience, Ohio, USA), 10µM each of forward and reverse primers, 1–2.5ng cDNA and molecular grade water to 12.5 µl. After an initial 10 min 95 °C enzyme activation step, 40 cycles of 95 °C for 15 s (step 1) followed by 60 °C for 30 s (step 2) were completed. Delta Ct (ΔCt, threshold cycle) was determined for each gene relative to the geometric mean of the housekeeping genes β-Actin and 18 S, and the well-established ΔΔCt method [16] was employed to enable comparisons between the groups.

### Immunofluorescence labelling of glia

**Fig. 1:**
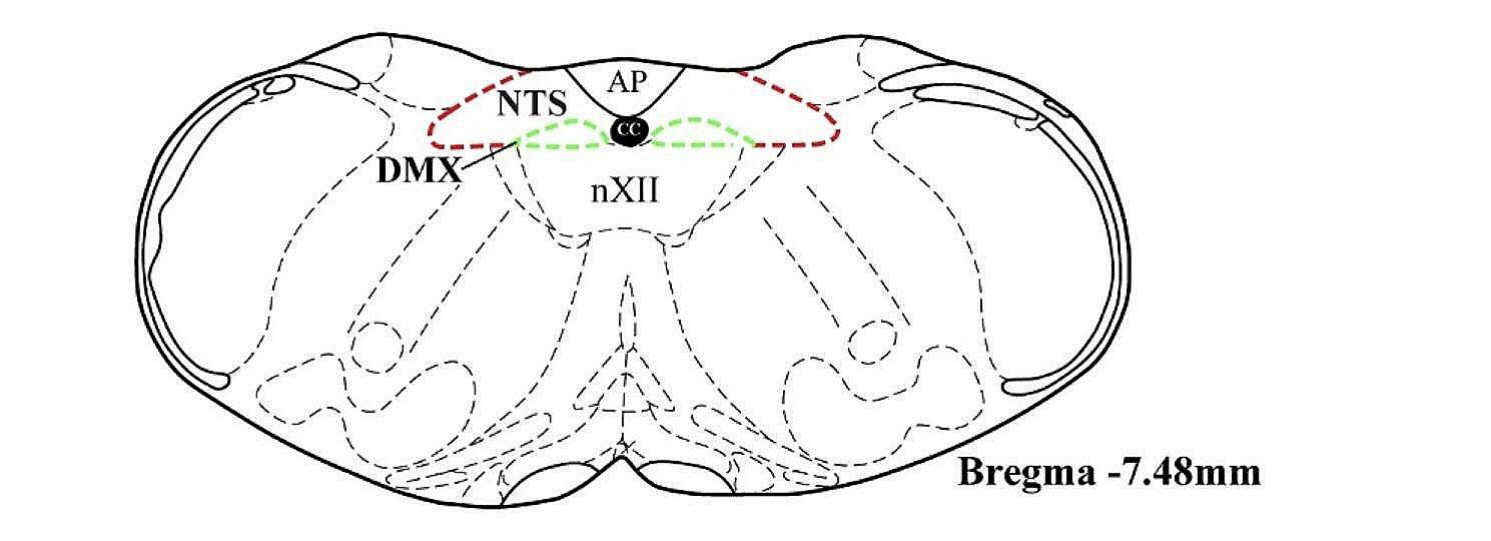
Brain atlas for identification of nuclei. Nuclei were identified using a brain atlas [17], at bregma level − 7.48 mm, with central canal (CC), area postrema (AP) and hypoglossal nucleus (nXII) for reference, with NTS outlined in red, DMX outlined in green

Brainstems (*n* = 6 per group) were dissected following euthanasia (i.p. sodium pentobarbital 150 mg/kg), and immersion-fixed in 4% paraformaldehyde (PFA) in phosphate buffer for 24 h then washed into phosphate buffered saline (PBS). Brainstems were then washed with 3 × 15 min 80% ethanol (EtOH), 3 × 15 min dimethyl sulfoxide (DMSO) and then 3 × 15 min 100% EtOH. The brainstems were then placed in a cell culture dish containing melted 1000 MW polyethylene glycol (PEG) in a 46 ^O^C vacuum oven (Thermoline, Wetherill Park, NSW) until the tissue had sunk (approximately 1 h). The tissue was then placed in a cryostat mould and embedded in 1450 MW PEG. Once hardened, the tissue block was sectioned at 30 μm on a rotary microtome. Every one in four sections from bregma level − 7.20 mm to -7.76 mm were taken and placed in PBS. Five sections were selected using a brain atlas [17], to ensure the correct region, and were then blocked in 10% normal donkey serum (NDS, Jackson ImmunoResearch) for 30 min. For labelling, sections were incubated at room temperature overnight in a solution of antibody diluent (0.3 M NaCl, 7.5mM Na2HPO4, 2.5mM NaH2PO4, 0.3% TritonX-1000, 0.05% Na Azide), 10% NDS, ionized calcium binding adaptor molecule 1 (Iba1; rabbit; 1:250; Wako; 019-19741) and glial fibrillary acidic protein (GFAP; chicken; 1:1000; Merck Millipore; AB5541). After the primary incubation, sections were rinsed in 3 × 15 min PBS and incubated for 2 h at room temperature with secondary antibodies Alexa594-donkey-anti-rabbit (1:50; Abcam; ab150076), Alexa488-donkey-anti-chicken (1:50; Jackson; JI703545155) and NeuroTrace Blue (1:25; ThermoFisher; N21479) in antibody diluent. Sections were mounted on slides in buffered glycerol and cover-slipped. Images were acquired using an Olympus BX50 microscope equipped with a mercury burner and an Olympus DP72 camera. Images were processed offline using ImageJ for mean fluorescent intensity (MFI) and percentage area (% area) quantification. The NTS and DMX, were selected using the neuronal marker NeuroTrace Blue and a mouse brain atlas for identification (Fig. 1) [17] and the same size area was taken for each image. To calculate MFI and % area for microglia and astrocytes, an image was converted into a 16-bit image, then duplicated and one image was converted to binary. The binary image was redirected to the 16-bit image and then the particle analysis was performed. An average MFI and % area were calculated for each animal, from the five sections taken. A custom MATLAB script was utilised to analyse microglia number and morphology [18–20]. This peer-reviewed script identifies the cell body, as determined by density of pixels, and then traces adjoining pixels from the cell body, again determined by their density. All images from a single nuclei were run at the same time to ensure consistency.

### Statistics

For weight data, statistical differences were determined using mixed-effect model, with Sidak post-hoc correction. For RT-qPCR and immunofluorescence, statistical differences were determined using a general linear model multivariate analysis of variance (ANOVA) or equivalent non-parametric test (Kruskal-Wallis with Dunn’s post-hoc) using SPSS with Sidak used for post-hoc correction. Significance set at *p* < 0.05. All graphs presented use untransformed means and standard errors of means (SEM) and created in GraphPad Prism v9 (GraphPad Software Inc., San Diego, CA, USA) and Adobe Illustrator 2023.

## Results

### Neonatal weight gain was significantly reduced due to infection

**Fig. 2:**
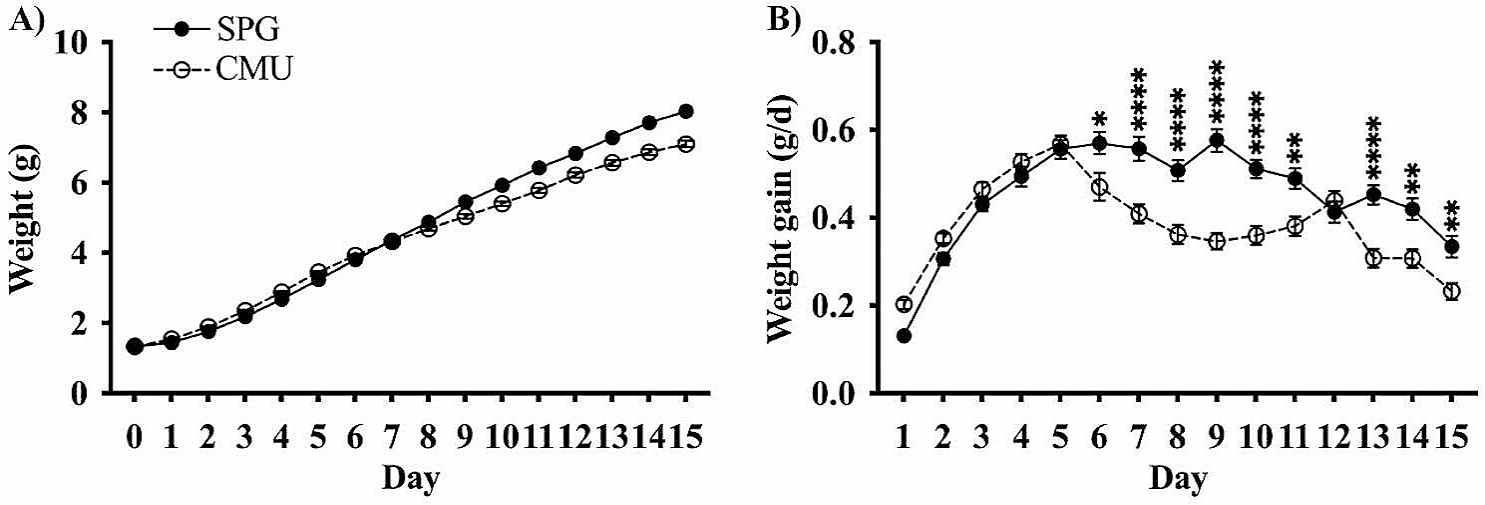
Acute effects of a neonatal respiratory infection on weight. The **A**) average gross weight and **B**) weight gain per day of SPG (solid line) and CMU (dotted line) infected mice, with significance between weight gain on a single day, SPG *n* = 27, CMU *n* = 28 (*p < 0.05, **p < 0.01, ****p < 0.0001)

Significant decreases in the rate of weight gain (Fig. 2B) of CMU infected mice compared to SPG infected mice were observed in neonates from days 6 to 15 (endpoint), with the exception of day 12, as shown in Fig. 2B. A significant decrease in weight gain is consistent with infection in mice.

### Confirmation of Chlamydia muridarum infection in the lungs

Along with the weight data to confirm infection, the expression of 16 S mRNA was measured at P15 in the lungs. The expression of a number of key inflammatory mediators were also quantified in the lungs to ensure that peripheral inflammation was present.

**Fig. 3:**
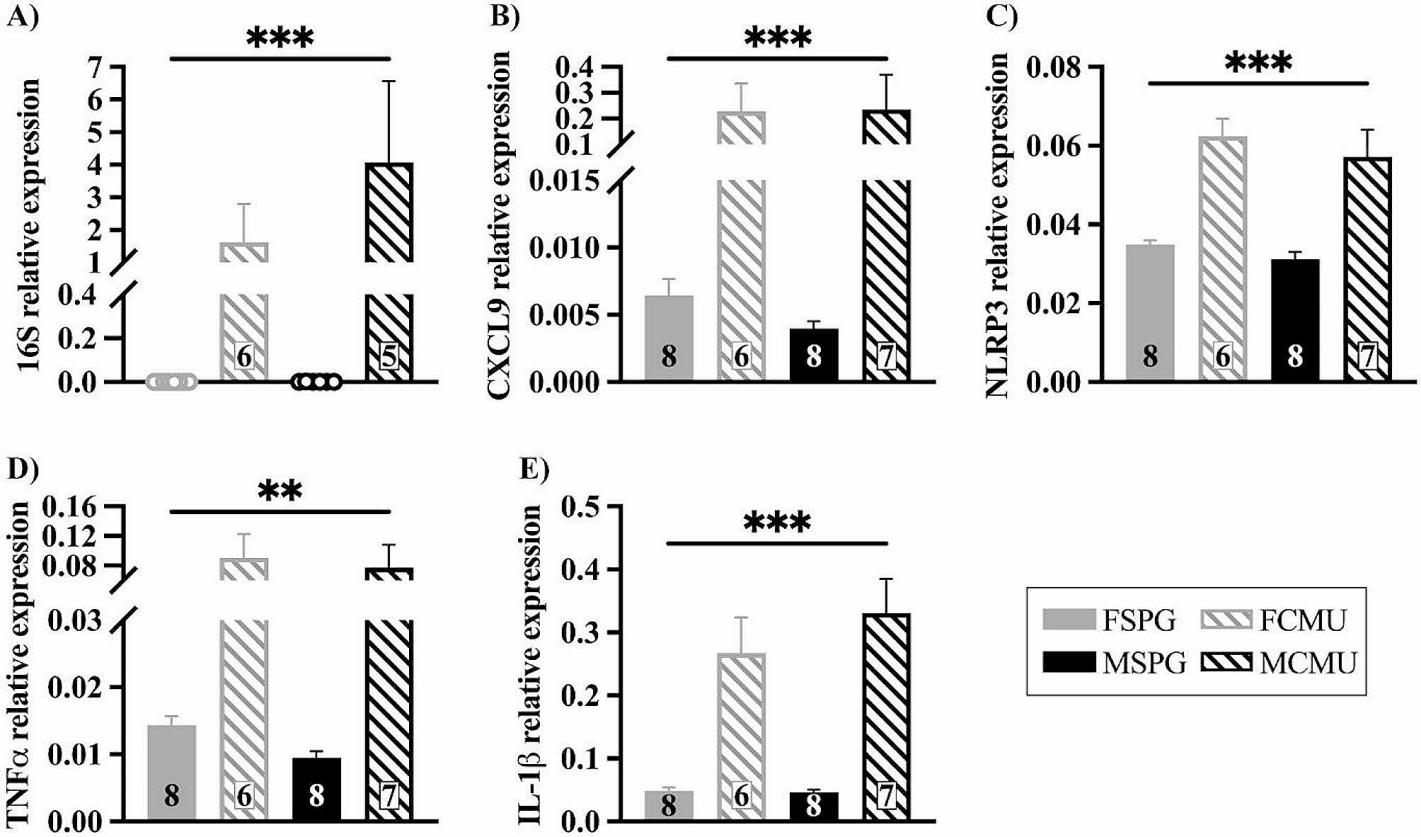
Upregulation in the expression of all inflammatory mediators in the lungs following neonatal infection. Graphs show gene expression levels (mean ± SEM) for SPG (solid bars) and CMU (hatched bars) females (grey) and males (black), with total number of samples shown in each bar. Inflammatory mediator expression; **A**) 16s (SPG vs. CMU ***p < 0.001), **B**) CXCL9 (SPG vs. CMU ***p < 0.001), **C**) NLRP3 (SPG vs. CMU ***p < 0.001), **D**) TNFα (SPG vs. CMU **p < 0.01) and **E**) IL-1β (SPG vs. CMU ***p < 0.001)

16 S is the central structural component of the bacterial subunit that is conserved in bacteria and allows identification of bacteria [21]. Given the variability in bacterial titre and clearance, a non-parametric test was performed, as 16 S expression data failed tests of homogeneity and Levene’s test of equal variances. There was a significant main effect of treatment, as shown in Fig. 3A, (treatment effect; H _(1,21)_ = 21.06, *p* < 0.001), this indicates that both male and female cohorts were successfully infected. Chemokine ligand 9 (CXCL9) is thought to aid in the infiltration and retention of activated T-cells in response to bacterial infection [22] and will give an indication of the direct response to the bacterial infection. There was a significant main effect in the expression of CXCL9 in the lungs at P15, shown in Fig. 3B (treatment effect; H _(1,25)_ = 21.85, *p* < 0.001). This indicates that the initial peripheral response to the bacterial infection is not sex-specific.

NLR family pyrin domain containing 3 (NLRP3) is a major component of the inflammasome and triggers the immune response. Once triggered, NLRP3 activates caspase-1 which then cleaves pro-interleukin 1β into its mature form, the a highly pro-inflammatory interleukin 1β (IL-1β) [23]. Tumour necrosis factor alpha (TNFα) is a pro-inflammatory cytokine produced during acute inflammation [24]. These mediators act together to initiate the inflammatory response in the lungs. At P15, there was a main effect of treatment on NLRP3, TNFα and IL-1β (treatment effect, NLRP3; F _(1,25)_ = 45.229, *p* < 0.001, TNFα; F _(1,25)_ = 13.235, *p* = 0.001, IL-1β; F _(1,25)_ = 51.409, *p* < 0.001) with CMU-infected mice having a significant increase compared to SPG-infected mice, as shown in Fig. 3C, D, E. There was no other main effects or interactions. This indicates that in the lungs there is a robust inflammatory response to infection that is not sex-specific.

### Neonatal infection alters inflammatory mediators in the medulla oblongata

This study examined the expression of a wide range of inflammatory mediators in the medulla oblongata in the acute phase of peripheral inflammation. Inflammatory mediators selected have been shown to be upregulated peripherally in response to infection, as well as mediators that a play crucial role in regulating the local immune environment of the CNS.

#### Upregulation of neuroprotective inflammatory mediators in infected females

**Fig. 4:**
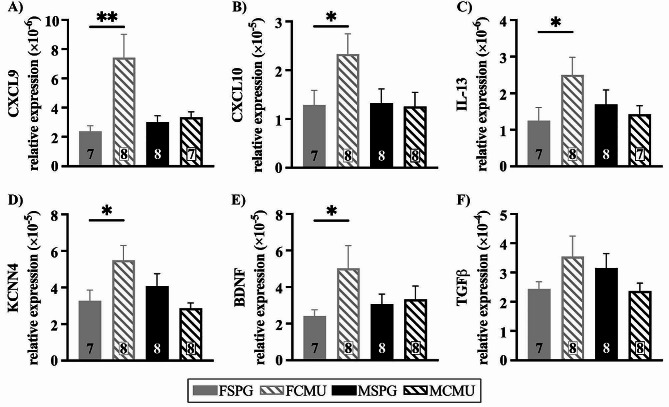
Upregulation of inflammatory mediators in the medulla oblongata of infected females. Graphs show **A**) CXCL9 (FSPG vs. FCMU **p < 0.01), **B**) CXCL10 (FSPG vs. FCMU *p < 0.05), **C**) IL-13 (FSPG vs. FCMU *p < 0.05), **D**) KCNN4 (FSPG vs. FCMU *p < 0.05), **E**) BDNF (FSPG vs. FCMU *p < 0.05) and **F**) TGFβ relative gene expression levels (mean ± SEM) at P15. Graphs presented as SPG (solid bars), CMU (hatched bars), females (grey) and males (black), with total number of samples shown in each bar

CXCL9 and chemokine ligand 10 (CXCL10) are two major chemokines that have been associated with immune cell migration, differentiation and activation [25]. Specifically, CXCL10 is primarily expressed on glia and recruits lymphocytes and phagocytes to inflamed areas to limit the spread of infection [26, 27] and as is the case with CXCL9, aids in T-cells responses to bacterial infection. These two chemokines will indicate the strength of the initial immune response to the bacteria. At P15 there was a significant treatment x sex effect in the expression of both CXCL9 and CXCL10 (treatment x sex, CXCL9; F _(1,26)_ = 6.682, *p* = 0.016, CXCL10; F _(1,27)_ = 5.472, *p* = 0.027). FCMU had a significant increase in the expression of both CXCL9 and CXCL10 compared to FSPG, as shown in Fig. 4A, B (post-hoc for females, CXCL9; F _(1,26)_ = 15.251, *p* = 0.001, CXCL10; F _(1,27)_ = 4.843, *p* = 0.036). There was no significant difference in MCMU compared to MSPG (post-hoc for males, CXCL9; F _(1,26)_ = 0.062, *p* = 0.805, CXCL10; F _(1,27)_ = 0.024, *p* = 0.879). These results suggest that females, unlike their male counterparts, are mounting a stronger response within the CNS to inhibit the development of infection during the acute phase.

Interleukin-13 (IL-13), functions in the CNS as an anti-inflammatory cytokine and is expressed by reactive microglia and can produce both beneficial and harmful effects on the local environment [28]. The IL-13 inflammatory pathway has been shown to modulate potassium calcium-activated channel subfamily N member 4 (KCNN4) which codes for K_Ca_3.1 [29]. K_Ca_3.1 is a voltage-independent potassium channel that is activated by intracellular calcium. Activation of this channel helps to sustain calcium entry into cells via voltage independent pathways and is critical for the inflammatory response in microglia and astrocytes [30, 31].

At P15, treatment x sex analysis revealed sex-specific effects of infection on both of these inflammatory mediators (treatment x sex, IL-13; F _(1, 26)_ = 5.820, *p* = 0.026, KCNN4; F _(1, 27)_ = 7.661, *p* = 0.010). For the expression of IL-13, there was significant difference between FCMU and FSPG, as shown in Fig. 4C (post-hoc for females; F _(1,26)_ = 5.281, *p* = 0.030) while there were no differences between males (post-hoc for males; F _(1,26)_ = 0.245, *p* = 0.624). Similarly, KCNN4 expression also showed significant differences between FCMU and FSPG, as shown in Fig. 4D (post-hoc for females; F _(1,27)_ = 6.192, *p* = 0.019) while there were no differences between males (post-hoc for males; F _(1,27)_ = 1.982, *p* = 0.171). This indicates that there are significant changes within the IL-13 inflammatory pathway in females only during the acute inflammatory response to a peripheral respiratory infection.

Brain derived neurotrophic factor (BDNF) is a crucial component in brain plasticity, neuronal development, growth and survival, and is implicated in establishing functional neuronal networks during development [32] .Transforming growth factor beta (TGFβ) is a multifactorial cytokine that regulates the behaviour of many immune cells, such as macrophages and neutrophils, causing anti-inflammatory effects and forming a network of negative immune regulatory inputs [33]. TGFβ signalling can regulate BDNF and both are considered protective when acutely responding to inflammation [34]. At P15, treatment x sex analysis revealed a significant effect of infection on BDNF expression (Fig. 4E), but no change to the expression of TGFβ (Fig. 4F) (treatment x sex, BDNF; F _(1, 27)_ = 5.235, *p* = 0.031, TGFβ; F _(1, 27)_ = 4.009, *p* = 0.055). The expression of BDNF in FCMU was significantly increased compared to FSPG, shown in Fig. 4E (post-hoc for females; F _(1,27)_ = 5.054, *p* = 0.033) and there was no significant difference in males (post-hoc for males; F _(1,27)_ = 0.056, *p* = 0.815). Together, this suggests that the initial potential protective response in the medulla oblongata is only seen in females, and it is driven by BDNF expression, not TGFβ.

#### Suppression of inflammatory mediators in infected males

**Fig. 5:**
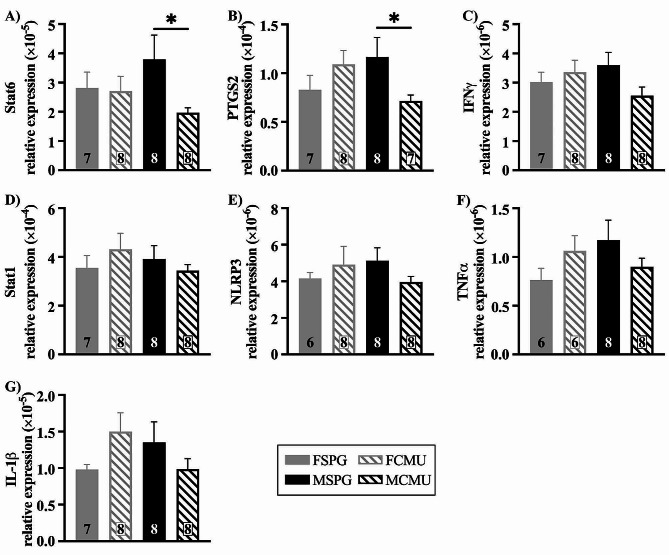
Downregulation of inflammatory mediators in the medulla oblongata of infected males. Graphs show **A**) Stat6 (MSPG vs. MCMU *p < 0.05), **B**) PTGS2 (MSPG vs. MCMU *p < 0.05), **C**) IFNγ, **D**) Stat1, E) NLRP3, F) TNFα and **G**) IL-1β relative gene expression levels (mean ± SEM) at P15. Graphs presented as SPG (solid bars), CMU (hatched bars), females (grey) and males (black), with total number of samples shown in each bar

Signal transducer and activator of transcription 6 (Stat6) is required for the promotion of the T-helper type 2 (Th2) immune response [35]. There was a significant treatment x sex interaction for Stat6 (treatment x sex, Stat6; F _(1, 27)_ = 5.831, *p* = 0.023). The expression of Stat6 showed a significant decrease in MCMU compared to MSPG, as shown in Fig. 5A (post-hoc for males; F _(1,27)_ = 5.395, *p* = 0.028) while there were no differences between females (post-hoc for females; F _(1,27)_ = 0.017, *p* = 0.896). This indicates that there are significant sex-specific changes, with infected males showing a suppression of potential anti-inflammatory pathways in the acute inflammatory response to a peripheral respiratory infection.

Prostaglandin-endoperoxide synthase 2 (PTGS2), also known as cyclooxygenase (COX-2), is a key enzyme in prostaglandin biosynthesis and during inflammation is associated with oedema and leukocyte trafficking [36]. Treatment x sex analyses revealed a significant effect on the expression of PTGS2 (Fig. 5B, treatment x sex, PTGS2; F _(1, 26)_ = 5.439. *p* = 0.028). MCMU showed a significant decrease compared to MSPG (post-hoc for males; F _(1,26)_ = 4.347, *p* = 0.047), with no differences seen in females (post-hoc for females; F _(1,26)_ = 1.472, *p* = 0.236). This indicates a clear suppression of male PTGS2 in response to the infection.

Interferon gamma (IFNγ) is a pro-inflammatory cytokine that primarily activates macrophages and stimulates neutrophils [37]. Signal transducer and activator of transcription 1 (Stat1) increases macrophage inflammatory responses [38] and is one of the primary transcription factors activated by IFNγ, thus playing a major role in immune responses [39]. However, at P15 no significant differences were seen in either IFNγ or Stat1, shown in Fig. 5C, D, indicating that, at this timepoint, this pathway is not heavily involved in the medulla oblongata’s initial response to a neonatal bacterial infection.

Given the importance of NLRP3, IL-1β, TNFα in the acute phase of the peripheral inflammatory response, the expression of these inflammatory mediators was also assessed in the medulla. However, there were no significant differences in NLRP3, TNFα or IL-1β, shown in Fig. 5E-G, indicating that, at this acute timepoint, this pathway is not heavily involved in the medulla oblongata’s acute response to a neonatal infection.

#### Glia in the respiratory centres of the brainstem are significantly affected by a neonatal respiratory infection

Both microglia, labelled with Iba1, and astrocytes, labelled with GFAP, were assessed in key regions of the medulla oblongata known to be important to the central processing of respiratory function. These are the nucleus tractus solitarii (NTS) and dorsal motor nucleus of the vagus (DMX).

#### NTS and DMX show significant alterations to microglia activation and morphology

**Fig. 6:**
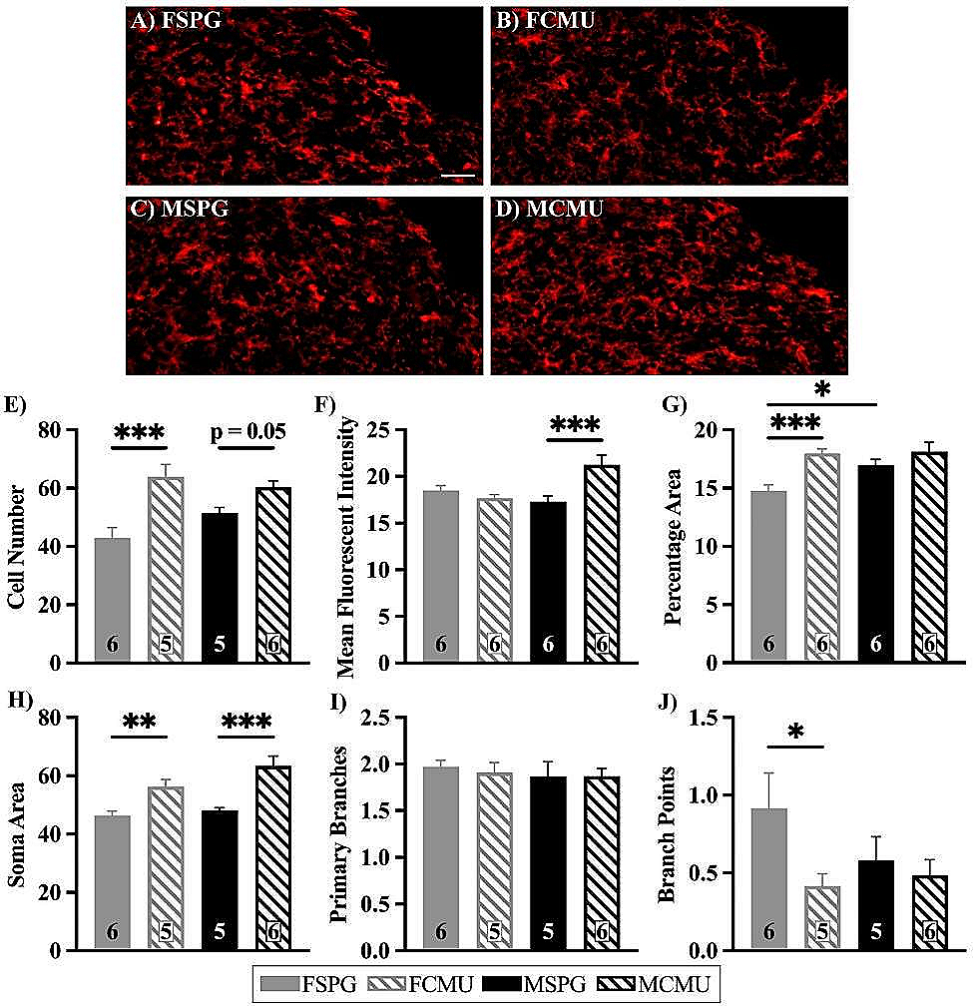
Iba1 immunofluorescent images and quantification of microglia in the NTS. Brainstem sections were labelled with Iba1 to identify microglia and nuclei were identified using a brain atlas. **A**-**D**) representative images of all groups showing the area selected for NTS with the area postrema removed, scale bars represent 100 μm. Graphs show **E**) number of cells (FSPG vs. MCMU ***p < 0.001), **F**) MFI (MSPG vs. MCMU ***p < 0.001), **G**) % area (FSPG vs. FCMU ***p < 0.001, FSPG vs. MSPG *p < 0.05), **H**-**J**) units in pixels, with **H**) soma area (FSPG vs. FCMU **p < 0.01, MSPG vs. MCMU ***p < 0.001), **I**) number of primary branches and **J**) number of secondary branches (branch points) (FSPG vs. FCMU *p < 0.05), (mean ± SEM). Graphs presented as SPG (solid bars), CMU (hatched bars), females (grey) and males (black), with total number of samples shown in each bar

In the NTS, there was a main effect of treatment on the number of cells (Fig. 6E; treatment effect; F _(1,18)_ = 23.946, *p* < 0.001). However, when this was further explored through treatment x sex analysis, a significant difference was only present between the number of cells in FCMU and FSPG (post-hoc for females; F _(1,18)_ = 23.815, *p* < 0.001), whereas males were approaching significance (post-hoc for males; F _(1,18)_ = 4.163, *p* = 0.056). This indicates that the magnitude of change to the number of cells differs between females and males. This sex-difference was mirrored in other analyses, with treatment x sex analysis revealing a significant difference in the mean fluorescent intensity (MFI) in males, where MCMU increased compared to MSPG, shown in Fig. 6F (treatment effect; F _(1,20)_ = 5.531, *p* = 0.029) (post-hoc for males; F _(1,20)_ = 17.410, *p* < 0.001). In contrast, females showed a significant difference in % area, with FCMU increasing compared to FSPG, shown in Fig. 6G (treatment effect; F _(1,20)_ = 15.109, *p* = 0.001) (post-hoc for females; F _(1,20)_ = 16.089, *p* < 0.001). Interestingly, treatment x sex analysis of microglia in the NTS revealed differences between the sexes in % area, with FSPG being smaller than MSPG, shown in Fig. 6G (post-hoc for SPG; F _(1,20)_ = 7.381, *p* = 0.013). Further morphological analyses were undertaken to examine the specific characteristics of the microglia. There was a main effect of treatment of soma area, Fig. 6H, (treatment effect; F _(1,19)_ = 29.554, *p* < 0.001). Again, when treatment x sex analysis was performed a magnitude difference was observed, with a greater difference observed between MCMU and MSPG (post-hoc for males; F _(1,18)_ = 21.919, *p* < 0.001, post-hoc for females; F _(1,18)_ = 9.039, *p* = 0.007). There was also a treatment x sex effect on the number of secondary branches (branch points), with FCMU decreasing compared to FSPG (treatment effect; F _(1,18)_ = 3.612, *p* = 0.073) (post-hoc for females; F _(1,18)_ = 5.076, *p* = 0.037), with no difference in males. Taken all together, the female’s response to infection results in more microglia, with larger somas and retracted branches, which accounts for the increase in % area. Whereas the male’s response to infection results in a bigger soma and a greater production of Iba1, as shown by the MFI. This indicates that while both female and male microglia are being affected in the acute phase of infection, the characteristics of the response to the insult differs.

**Fig. 7:**
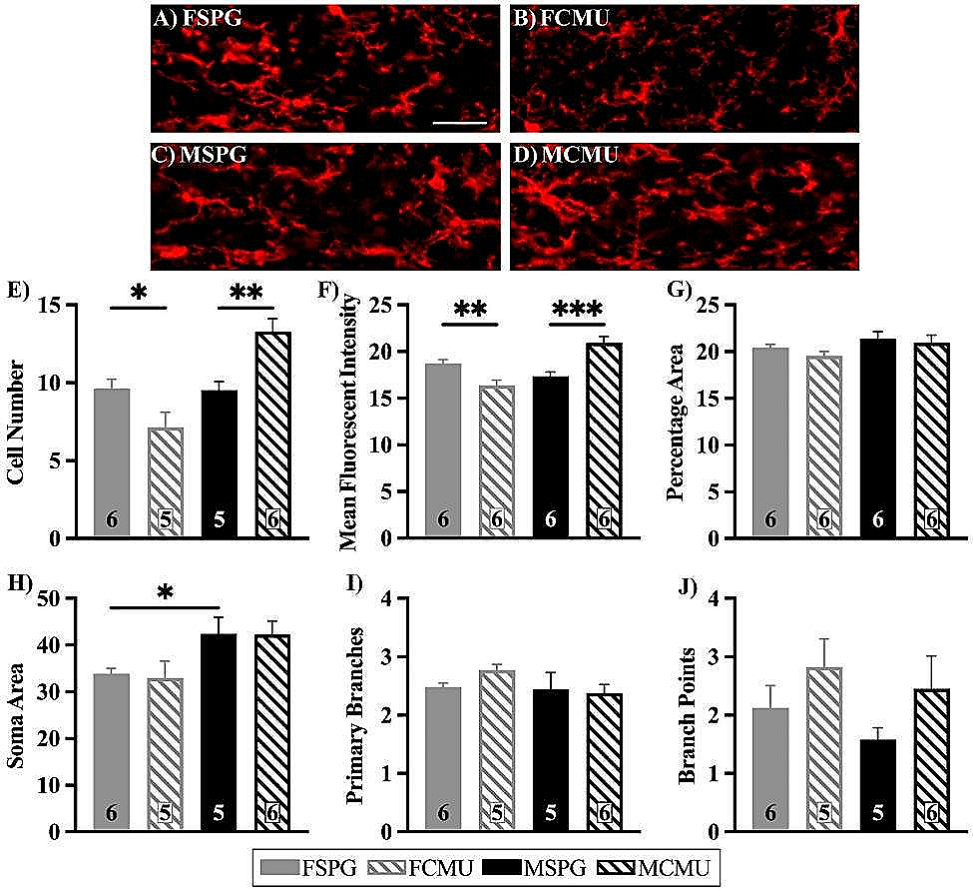
Iba1 immunofluorescent images and quantification of microglia in the DMX. Brainstem sections were labelled with Iba1 to identify microglia and nuclei were identified using a brain atlas. **A**-**D**) representative images of all groups showing the area selected for DMX, scale bars represent 100 μm. Graphs show **E**) number of cells (FSPG vs. FCMU *p < 0.05, MSPG vs. MCMU **p < 0.01), **F**) MFI (FSPG vs. FCMU **p < 0.01, MSPG vs. MCMU ***p < 0.001), **G**) % area, **H**-**J**) units in pixels, with **H**) soma area (FSPG vs. MSPG *p < 0.05), **I**) number of primary branches and **J**) number of secondary branches (branch points) (mean ± SEM). Graphs presented as SPG (solid bars), CMU (hatched bars), females (grey) and males (black), with total number of samples shown in each bar

In the DMX, treatment x sex analysis (treatment x sex, F _(1, 18)_ = 17.458, *p* = 0.001) revealed there was a significant decrease in the cell number of FCMU compared to FSPG, shown in Fig. 7E (post-hoc for females; F _(1,18)_ = 5.517, *p* = 0.030) while there was a significant increase in cell number of MCMU compared to MSPG (post-hoc for males; F _(1,18)_ = 12.674, *p* = 0.002). This was reflected in the analysis of MFI (treatment x sex, F _(1, 20)_ = 30.167.

*p* < 0.001), with a significant increase in the MFI between MCMU and MSPG, shown in Fig. 7F (post-hoc for males; F _(1,20)_ = 22.062, *p* < 0.001), whereas in females, FCMU saw a significant decrease compared to FSPG, Fig. 7F (post-hoc for females; F _(1,20)_ = 9.428, *p* = 0.006). Unlike the NTS, morphological analyses of the DMX showed no significant effect of infection on any characteristic, indicating in the acute phase a respiratory infection only impacts the number of microglia present in the DMX. Interestingly, sex x treatment analysis revealed differences between the sexes in soma area, with FSPG having a smaller soma than MSPG, shown in Fig. 6H (sex effect; F _(1,18)_ = 9.810, *p* = 0.006) (post-hoc for SPG; F _(1,18)_ = 4.513, *p* = 0.048). This indicates a different starting point between male and female microglia regardless of environmental factors.

#### Astrocyte changes are minimal following neonatal respiratory infection

**Fig. 8:**
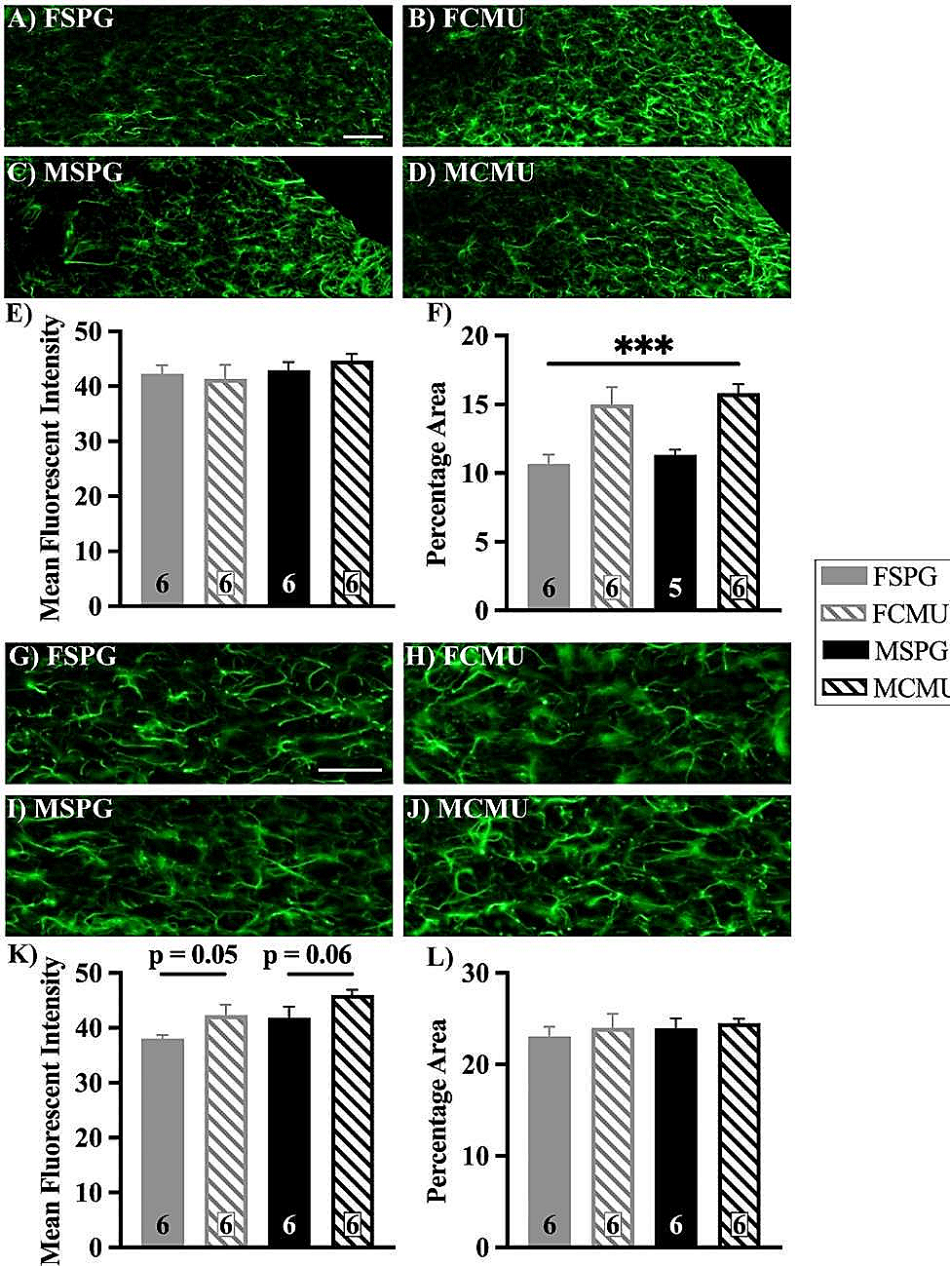
GFAP immunofluorescent images and quantification of astrocytes in the NTS and DMX. Brainstem sections were labelled with GFAP to identify astrocytes and nuclei were identified using a brain atlas. **A**-**D**) representative images of all groups showing the area selected for NTS with the area postrema removed, **G**-**J**) representative images of all groups showing the area selected for DMX, scale bars represent 100 μm. Graphs show **E**) MFI and **F**) % area (SPG vs. CMU ***p < 0.001) in the NTS and **K**) MFI and **L**) % area in the DMX (mean ± SEM). Graphs presented as SPG (solid bars), CMU (hatched bars), females (grey) and males (black), with total number of samples shown in each bar

There was a main effect of treatment (treatment effect, F _(1, 19)_ = 27.138, *p* < 0.001) on the % area of NTS GFAP + ve cells, shown in Fig. 8F. This indicates that astrocytes from both sexes show similar responses to infection. There were no changes in either MFI of the NTS or either parameter within the DMX.

## Discussion

It is well known that the critical period of development is particularly susceptible to environmental stressors, however the specific effect within brainstem respiratory centres remains unknown. In addition, the majority of data that is available explores the response to a bacterial mimetic (like lipopolysaccharide [LPS]) and does not investigate the more complex response from exposure to the entire bacterium [40]. This study explores the acute effects of a neonatal respiratory infection on the brainstem centres that are essential for respiratory control. There was a significant decrease in the rate of weight gain of the CMU-infected mice as well as a significant increase in inflammatory mediators in the lungs that were not sex-specific, indicating that there was a robust inflammatory response to a respiratory bacterial infection in the lungs. In the brainstem, however, inflammatory mediators showed a strong sex-specific response, with infected females showing increases in neuroprotective inflammatory mediators and males showing decreases in a few inflammatory mediators. There were also significant effects on microglia in both males and females. In the DMX, infected females observed to have a decrease in the MFI and microglia cell number, and a contrasting impact in infected males of a significant increase in MFI and cell number. Astrocytes demonstrated a limited acute response, with a significant increase in percentage area only occurring in the NTS of both sexes. Overall, these data indicate that a neonatal respiratory bacterial infection not only produces a robust response in the lungs but also a strong sex-specific response in the brainstem centres that control respiration. This infection and subsequent inflammation during a critical period of development of the CNS could interrupt key developmental processes, and lead to life-long dysfunction.

### The peripheral response to a neonatal respiratory infection

The peripheral response in this model has been well characterised and established in female mice [13]. To identify any sex-specific peripheral changes and to validate that the effects seen in the brainstem are related to infection of the lungs, infection and the peripheral inflammatory response had to be confirmed. There was a significant reduction in weight gain in the CMU-infected animals from days 6 until endpoint (day 15/2wks). There was also saw a significant upregulation of 16 S expression in both FCMU and MCMU at the endpoint (day 15/2wks). This mirrored previous work from our laboratory showing a significant reduction in weight gain from days 7 to 23 in response to infection, with peak of infection at day 12 and peak of inflammation at day 15/2wks in the lungs [13]. The reduction in weight gain is indicative of infection in the pups, and coupled with the 16 S expression, successfully confirms infection in this cohort. To ensure there had been no drift in the model in terms of inflammatory response, key inflammatory mediators were examined in the lungs, IL-1β, NLRP3, TNFα and CXCL9. All mediators showed a significant increase in the CMU-infected mice, regardless of sex, indicating a robust inflammatory peripheral phenotype that is consistent with our previous findings.

### Sex-specific inflammatory mediator response in the medulla oblongata

A key finding of this study is that, while a neonatal respiratory infection caused the same peripheral inflammatory response in both males and females, sex did have a significant impact on the inflammatory response within the brainstem centres, housed in the medulla oblongata, that control respiration.

With respect to the female response to infection-induced neuroinflammation, there was significant upregulation in the expression of CXCL9, CXCL10, BDNF, IL-13 and KCNN4 in the medulla oblongata. In the acute response phase these inflammatory mediators are all considered to be neuroprotective, with the exception of KCNN4. Both CXCL9 and CXCL10 control the spread of infection in the CNS through the recruitment of effector cells, such as phagocytes and lymphocytes, to the CNS. This has been shown in other infections in adult models, such as viral infections; herpes simplex virus [41] and the West Nile virus [42], where it was linked to reductions in mortality following infection. BDNF is well-established as a key factor in neuronal development and plasticity and has been shown to reduce inflammation and neuronal apoptosis in response to bacterial infections [43]. Early-life deficiency of BDNF has been linked to several mood disorders such as depression [44, 45] and bipolar disorder [46]. Therefore, this increase in females, in response to the early life infection, could protect the CNS from inflammation induced damage. Although, IL-13 is a major driver of allergic asthma in the lungs [47], in the CNS it has anti-inflammatory effects and has been shown to provide neuroprotection through anti-inflammatory microglial responses in stroke [48] and to promote recovery after a traumatic brain injury, again through microglia-mediated immune responses [49]. KCNN4 is the only upregulated mediator that would be considered damaging to the CNS. KCNN4 contributes the pro-inflammatory activation of microglia [50], with blocking of this channel shown to improve CNS outcomes following traumatic brain injury [51, 52] and has been proposed as a potential therapeutic for microglia neurotoxicity in Alzheimer’s disease [53]. Given the response in this study of the microglia in females, i.e., decreased numbers and MFI (discussed further below), KCNN4 appears to be having only a limited effect or the system is plastic enough to overcome these alterations. Overall, at the 2wk timepoint in the medulla, there is a strong anti-inflammatory effect in females, which would likely protect the CNS from damage caused by the peripheral inflammatory event.

Unlike the similar peripheral response observed in the lungs. the response of the medulla oblongata in males to a peripheral inflammatory event is contrasting and significantly muted compared to females. There was a significant decrease in only two inflammatory mediators, PTGS2 and Stat6, in response to the respiratory infection, with no changes to any other mediator observed. PTGS2 plays a central role in the inflammatory cascade of both anti- and pro-inflammatory mediators [54]. Multiple studies have reported that by either inhibiting or knocking down PTGS2, the CNS response to a systematic inflammatory challenge is exacerbated by increasing microglia activation and permeability of the BBB to infiltrating peripheral immune cells [55–57]. However, these studies have been conducted in adult models of systemic inflammation and use LPS, a bacterial mimetic, to induce inflammation. Our recent work exploring the brainstem’s response to neonatal LPS inflammation begins to fill in this gap in knowledge [58], demonstrating a reduction in PTGS2 in males during the acute response and that overall, the response to neonatal LPS followed similar trends to CMU, with suppression of inflammatory mediators in males. There is no information surrounding a specific neonatal respiratory infection and the response of PTGS2 in the medulla at the neonatal age. Stat6 helps promote microglial clearance of dying cells which is key to the resolution of neuroinflammation. Supressing Stat6 has been shown to exacerbate local inflammation and worsen long-term outcomes [59, 60]. Again, these studies were completed in adult models and the neonatal response could differ, but there is no literature available for this early postnatal age. It is well described in clinical literature that male neonates are more susceptible to infection due to higher testosterone levels supressing the immune system [61–63]. Suppression at this age does not allow the system to correctly respond and clear the infection, causing increased mortality as well as an increased likelihood of severe long-term health consequences in males.

The differences between the strength of the central and the peripheral response could relate to timing. The CNS response to a peripheral infection is known to be slower than in the periphery itself [64], therefore this would suggest that at the 2wk timepoint, we are observing the emerging response within the brainstem to the peripheral respiratory infection. This would explain the strong upregulation of pro-inflammatory mediators in the lungs in both sexes, contrasted with the variable shift in the pro-inflammatory mediators in the medulla. Since only one timepoint was observed within this acute inflammatory phase, it remains to be seen whether in females, the significant neuroprotective changes are the initial response to peripheral infection-induced neuroinflammation or if this a counter measure to reduce a previous peak in the pro-inflammatory response. The same could be said for males, with the trends towards suppression of inflammatory mediators potentially being indicative of either the start or the tapering off of the neuroinflammatory response. To determine which of these is true, the medulla’s response to peripheral infection induced neuroinflammation requires a more comprehensive exploration throughout this acute inflammatory phase.

### Glial response to neonatal respiratory infection

The acute response of microglia and astrocyte to a peripheral respiratory infection was nuclei- and sex-specific. Activation appeared to be enhanced in males and suppressed in females, with further, differential changes observed between the NTS and DMX, with significant alterations to microglial morphology and cell number in the NTS and changes to cell number in the DMX. In females, the number of microglia in the DMX decreased in response to a respiratory infection, further indicating a strong anti-inflammatory neuroprotective phenotype. Suppression of the female microglia response in the DMX could be attributed to the increases observed in anti-inflammatory immune mediators, mainly IL-13, which has been shown to reduce neuroinflammation through induction of microglia apoptosis [65]. The morphological changes in the NTS suggest a neuroinflammatory phenotype for both males and females, which could lead to long-term consequences. At this age, neonatal microglia have a very similar function to adult microglia but have a distinct phenotype. Adult microglia are transient in their nature, whereas neonatal microglia exhibit a more classically defined amoeboid morphology, making the neonatal period a critical window for microglial development [66]. Activation of neonatal microglia via a peripheral infection, and subsequent neuroinflammation, can result in persistent changes in neonatal microglial function which then remains into adulthood [67]. This alteration has been linked to neurodevelopment disorders [68] and respiratory deficits [69] in adulthood. The significant sex-specific responses to infection and the baseline sex differences observed in the soma area of the DMX, does indicate that male and female microglia have a distinct phenotype. This is well characterised in the literature with the sex differences believed to be derived from large increase in male gonadal hormones in males and the sex chromosome complement [70]. In addition, other studies have found that oestrogen receptors on microglia are undetectable in the early life stages [71], which does indicate alternate mechanisms for sex-specific effects. As the NTS modulates and disseminates afferent sensory information and the DMX sends efferent information, the different changes to morphology and number of microglia would have a distinct impact on signalling both centrally and in the periphery.

Astrocytes showed an increase in % area, in the NTS only, in both males and females and thus was not sex-specific. The marker used in this study, GFAP, plays a role in supporting BBB function and is elevated with astrocyte activation [72]. Astrocyte activation can lead to thickening and elongation of astrocyte processing that would be indicated through altered percentage area [73]. The NTS is the primary integration centre for all incoming peripheral signals from the vagus nerve, including a large component from the lungs, and contributes to synaptic transmission through gliotransmission and regulation of glutamate [74, 75]. Thus, it is not surprising at this acute stage, astrocytes within the NTS show the greatest response as this is the first integration centre to receive the peripheral inflammatory signals and then further distribute these signals around the CNS. This further emphasises the need for a deeper exploration of this acute inflammatory phase, including ages either side of this P15 time point, to more accurately assess the early response of astrocytes to a neonatal respiratory infection.

### Development consequences of a neonatal respiratory infection

During early postnatal development, neurons associated with respiration undergo maturational changes which are crucial to ensuring the proper functioning of the respiratory system. While the fundamental processes of these neurons are established at birth, postnatal fine tuning of the system allows many tightly regulated processes to develop to allow rapid and effective responses to environmental demands. In rodent studies, the DMX has been shown to undergo postnatal maturation in neuronal function, with markers of neuronal activation revealing a plateau in activity occurring at days 3–4 and 12 [76]. Further study by McMenamin et al. demonstrated that during the first postnatal week DMX neurons received inhibitory currents from both gamma-aminobutyric acid (GABA) and glycine sources, which are both fast currents during this developmental period. By the second postnatal week, there was a switch to only GABAergic inhibitory currents that gradually slowed into adulthood [77]. GABA has known interactions with inflammatory processes [78, 79], therefore, disruption to this developmental switch by neonatal inflammation could potentially alter the profile of these currents, leading to hypersensitivity of the neuronal respiratory circuit and adversely affecting vagal efferent control of the respiratory system.

Similar maturation processes have been described for the development of NTS neurons, with a downregulation of BDNF at day 12 [80]. This normal developmental reduction leaves the system susceptible to stressors during this critical window, and given that this Chlamydia model results in a peak of inflammation at day 12, this could be altering a key developmental trajectory of neurotransmission within the respiratory circuit. In this study, we do see an increase in BDNF in females, however the long-term changes still need to be assessed. Other normal developmental processes of NTS neurons include increases to synapse formation and decreases to membrane resistance over the first four postnatal weeks [81]. This maturation of NTS neurons renders NTS neurons less sensitive to inputs and represents refinement of brainstem circuitry associated with respiration. However, disruption to this developmental refinement by a neonatal respiratory infection could lead to dysfunction within these processes, again resulting in a hypersensitivity of the system, and highlights the vulnerability of the system to a neonatal respiratory infection.

### Limitations and future directions

This is one of the first studies to evaluate the acute effects of a neonatal respiratory infection on the brainstem. However, there are specific limitations which need to be investigated in future studies. The medulla oblongata contains multiple nuclei with a diverse array of functions. Since the RNA for the expression of inflammatory mediators in this study was extracted from the entire medulla, conclusions cannot be drawn upon its specificity to individual nuclei, nor the relative inflammatory response within other regions of the brainstem. In future RT-qPCR studies, individual nuclei should be isolated to detail each specific nuclei’s inflammatory response. Another limitation is while a link is hypothesised between the respiratory infection and the medulla inflammatory response, this needs to be explored functionally. To validate this hypothesis, knock-out models can be generated to eliminate key neuroinflammatory pathways and functionally assess the respiratory response under these conditions.

## Conclusions

This study clearly demonstrates that a neonatal respiratory infection induces acute sex-specific neuroinflammation in the respiratory centres of the medulla oblongata, which does not directly mirror the inflammatory response in the periphery. In females, there was a significant neuroprotective phenotype displayed with increased anti-inflammatory immune mediators and a suppression of microglia activation within the DMX only. Conversely, in males, there was suppression of inflammatory mediators and activation of microglia which could ultimately lead to worse outcomes. The neonatal period is a critical window for development and disruption during this time can lead to significant neurodevelopmental challenges and long-term pre-disposition to further damage. Given these centres control major components of respiration, neuroinflammation within these regions could lead to major respiratory deficits with outcomes such as sudden infant death syndrome [82], asthma [5] and sleep apnoea [83].

## Data Availability

The data sets used and/or analysed during the current study are available from the corresponding author on reasonable request.
